# Effect of nano-hydroxyapatite coating on the osteoinductivity of porous biphasic calcium phosphate ceramics

**DOI:** 10.1186/1471-2474-15-114

**Published:** 2014-04-01

**Authors:** Jianzhong Hu, Yongchun Zhou, Lihua Huang, Jun Liu, Hongbin Lu

**Affiliations:** 1 Department of Spine Surgery, Xiangya Hospital, Central South University, Changsha, 410008, China; 2 Center for Medical Experiments, Third Xiangya Hospital, Central South University, Changsha, 410013, China; 3 Department of Sports Medicine, Research Center of Sports Medicine, Xiangya Hospital, Central South University, Changsha, 410008, China

**Keywords:** Mesenchymal stem cell, Cell proliferation, Osteogenesis, Bone regeneration, Biocompatibility, Nanotopography

## Abstract

**Background:**

Porous biphasic calcium phosphate (BCP) ceramics exhibit good biocompatibility and bone conduction but are not inherently osteoinductive. To overcome this disadvantage, we coated conventional porous BCP ceramics with nano-hydroxyapatite (nHA). nHA was chosen as a coating material due to its high osteoinductive potential.

**Methods:**

We used a hydrothermal deposition method to coat conventional porous BCP ceramics with nHA and assessed the effects of the coating on the physical and mechanical properties of the underlying BCP. Next, its effects on mesenchymal stem cell (MSC) attachment, proliferation, viability, and osteogenic differentiation were investigated.

**Results:**

nHA formed a deposited layer on the BCP surface, and synthesized nHA had a rod-like shape with lengths ranging from ~50–200 nm and diameters from ~15–30 mm. The nHA coating did not significantly affect the density, porosity, flexural strength, or compressive strength of the underlying BCP (*P* > 0.1). Scanning electron microscopy showed MSC attachment to the scaffolds, with a healthy morphology and anchorage to nHA crystals via cytoplasmic processes. The densities of MSCs attached on BCP and nHA-coated BCP scaffolds were 62 ± 26 cells/mm^2^ and 63 ± 27 cells/mm^2^ (*P* > 0.1), respectively, after 1 day and 415 ± 62 cells/mm^2^ and 541 ± 35 cells/mm^2^ (*P* < 0.05) respectively, after 14 days. According to an MTT assay, MSC viability was higher on nHA-coated BCP scaffolds than on BCP scaffolds (*P* < 0.05). In addition, MSCs on nHA-coated BCP scaffolds produced more alkaline phosphatase, collagen type I, and osteocalcin than MSCs on BCP scaffolds (*P* < 0.05).

**Conclusions:**

Our results demonstrate that BCP scaffolds coated with nHA were more conducive for MSC adhesion, proliferation, and osteogenic differentiation than conventional, uncoated BCP scaffolds, indicating that nHA coating can enhance the osteoinductive potential of BCP ceramics, making this material more suitable for applications in bone tissue engineering.

## Background

Bone defects induced by trauma, surgery, or tumor resection are very common and thus consume a large amount of medical resources annually. About 9 million fragility fractures occur worldwide every year [1]. In 2005, in the United States alone, 1 million trauma fracture patients required hospitalization, 300,000 cases of spinal fusion surgery cost more than $20 billion in medical resources, and treatment of more than 3,000 pediatric patients with bone cancer cost more than $70 million [2]. Autogenous bone grafting is the gold standard for treating bone defects, but sources of donor tissue are limited. Furthermore, autologous bone grafts have shown considerable bone resorption before bone healing [3,4]. Bone allografts are not extensively applied in clinical practice due to the associated risks of infection and immune rejection [5,6]. Therefore, synthetic materials have been studied extensively as potential bone substitutes, including calcium phosphate (CaP) ceramics [7-9]. One CaP ceramic material, biphasic calcium phosphate (BCP), is composed of hydroxyapatite (HA) and beta-tricalcium phosphate (β-TCP) and exhibits good biocompatibility and bone conduction performance [10,11]. In addition, BCP degradation and biological activity can be controlled by adjusting the ratio of HA to β-TCP [11,12]. However, due to a lack of inherent osteoinductivity [11,13], limited formation of new bone occurs after osteoconduction is achieved, and thus, conventional BCP often requires the additional use of osteoinductive biomolecules (e.g., bone morphogenetic proteins) for enhanced osteoinduction [9-11,14].

In recent years, nanotechnology has been applied to the development of CaP ceramics for bone regeneration [15]. It was discovered that human bone apatite is composed of nanosized CaP crystals and that nano-hydroxyapatite (nHA) is similar to the inorganic component of human bone [9,16]. A nHA surface, such as the previously applied nHA particles, nHA-coated nHA/gelatin, nHA-coated roughened titanium, can not only promote the adhesion, proliferation, and osteogenic differentiation of bone-related cells and improve mineral deposition [17-21], but also can improve the biocompatibility, biological activity, and bone integration ability of the underlying biomaterial [20-22]. In addition, compared to conventional BCP ceramics, those with a nanostructured surface have greater osteoinductive potential [9,23]. Considering these findings along with the interconnected porous structure of human bone [16], the present study aimed to improve upon existing bioceramic materials by coating porous BCP ceramics with nHA to form a new bioresorbable ceramic scaffold that simulates the chemical characteristics and physical structure of human bone on both the macro-and micro-scales.

As a potentially unlimited source of donor tissue, stem cells offer immense promise for bone repair and regeneration [24], and mesenchymal stem cells (MSCs) can be induced to differentiate into bone, amongst other tissues [25]. MSCs can be harvested from a patient’s bone marrow, expanded in culture, induced to differentiate, and seeded on a biomaterial scaffold prior to implantation into a bone defect [24,26,27]. MSCs have been widely used to test potential scaffolds formed from various biomaterials, such as calcium phosphate cement [24], hydroxyapatite [17], and silk gland fibroin protein [26]. In the present study, we compared the osteogenic differentiation of MSCs seeded on our nHA-coated porous BCP scaffolds as well as on conventional BCP scaffolds. We hypothesized that the nHA coating on porous BCP ceramics would promote the proliferation, viability, and osteogenic differentiation of MSCs, thus demonstrating the osteoinductivity of this new biomaterial scaffold.

## Methods

### Preparation of nHA-coated, porous BCP ceramics

Clinical grade porous BCP ceramics were produced by Gong-Chuang Biofunctional Materials Co., Ltd, Hengyang, China. The specimens used for cell studies were discs of 11 mm in diameter and 2 mm in thickness, those for the flexural strength testing were 5 × 8 × 25-mm cuboids, and those for compressive strength testing were 5 × 5 × 12.5-mm cuboids. All specimens were polished with SiC sand paper (1500 grit), washed with anhydrous ethanol for 30 minutes in an ultrasonic cleaner, and then rinsed with deionized water prior to use.

A hydrothermal deposition method was applied to prepare nHA for coating, using analytical grade reagents purchased from Alfa Aesar. First, 0.3 mol/L ammonium dihydrogen phosphate and 0.5 mol/L nitrate tetrahydrate calcium solution were prepared in deionized water. Then 20 g PVP (polyvinylpyrrolidone) was added to the ammonium dihydrogen phosphate solution, which was heated with stirring until the PVP was completely dissolved, and after the pH value of the solution had been adjusted to 11 with concentrated aqueous ammonia, BCP was added to the solution. Calcium nitrate tetrahydrate solution was added under ultrasonic irradiation at a rate of 2 mL/min into the above reaction mixture, with continued ultrasound for 30 minutes, and the resulting mixture was transferred to the hydrothermal synthesis reactor (Qiang-Qiang Instruments, Shanghai, China) and maintained under hydrothermal conditions at 120°C for 12 hours. After completion of the reaction, the reaction vessel was naturally cooled to room temperature, and then the nHA-coated, porous BCP ceramic samples were removed, filtered, washed with anhydrous ethanol and distilled water alternately, and dried at room temperature before being ready for use.

### Microstructure characterization

After completion of the reaction, the precipitate was washed completely with ethanol to remove residual PVP molecules, collected, and dried at 60°C in a vacuum oven. Then a small amount of precipitate was ultrasonically dispersed in anhydrous ethanol, and a drop was taken and dripped onto an ultra-thin carbon film. After drying, the size and shape of the nHA were observed using field emission transmission electron microscopy (FETEM, JEM-2100 F, JEOL, Tokyo, Japan), and the morphology of the coating and pore size distribution on the material surface and section pore wall were observed using field emission scanning electron microscopy (FESEM, Nova NanoSEM 230, FEI Co., Hillsboro, OR, USA). For microstructure characterization, five view fields were examined on each of 12 samples for each specimen (60 fields in total). The phase compositions of the BCP and nHA-coated BCP surfaces were characterized using X-ray diffraction (XRD, D/ruax 2550PC, Rigaku, Japan).

The density and porosity of the specimens were measured according to Archimedes’ method. First, the specimen was dried in a vacuum oven at 60°C for 24 hours to determine the dry weight (W_1_). Then, the specimen was transferred to a beaker and placed in a vacuum oven at 2 kPa for 15 minutes, followed by slow injection with water until the specimen was completely immersed. Next, the pressure was gradually restored to atmospheric, and then the saturated specimen was placed in a copper wire basket, suspended in a beaker filled with water, and weighed (W_2_). The specimen was taken out of the water, any water remaining on its surface was removed with wet gauze, and the specimen was weighed again (W_3_). The porosity was calculated using the following formula: porosity (%) = [(W_3_ – W_1_) / (W_3_ – W_2_)] × 100%. The density was calculated using the following formula: density (g/cm^3^) = [W_1_/(W_3_ – W_2_)] × d, where d is the density of water (1 g/cm^3^).

### Characterization of the mechanical properties

The compressive strength of the specimens was tested using a universal testing machine (Dual Column Testing System 3369, Instron Co., Norwood, MA, USA) with a crosshead speed of 0.2 mm/min. Compressive strength (*C*) was calculated by *C = P/A*, where *P* is the critical load and *A* is the specimen cross-sectional area [28]. The flexural strength of the specimens was measured by the three-point flexural method with a span of 20 mm at a crosshead speed of 0.5 mm/min. Flexural strength (*S*) was calculated by *S* = 3 *FL*/2*bd*^2^, where *F* is the maximum load, *L* is flexure span, *b* is specimen width, and *d* is specimen thickness [29]. For each material, 12 specimens were tested to determine these mechanical properties.

### Isolation and culture of bone marrow MSCs

MSCs were harvested from the bone marrow of 4-week-old New Zealand rabbits, using an extraction method similar to that described in Lim et al. [30] and Ouyang et al. [31]. The bilateral posterior iliac crests of anesthetized rabbit were disinfected, and 4 ml bone marrow was extracted from the bilateral iliac crests using a puncture needle with saline containing 1000 U/ml sodium heparin and mixed with heparin. The anti-coagulated bone marrow was diluted with 5 times the volume of low glucose Dulbecco’s Modified Eagle Media (DMEM, Gibco, Rockville, MD, USA). The bone marrow mixtures were centrifuged by Percoll density gradient at 1800 rpm for 20 minutes at room temperature, and the nucleated cell layer in the middle was carefully drawn out, added to the DMEM, and cleaned by two 10-minute rounds of 1200 r/min centrifugation. The bone marrow nucleated cells were harvested and then resuspended in control media [DMEM supplemented with 10% fetal bovine serum (FBS, Gibco) and 1% penicillin/streptomycin (Sigma Aldrich, St. Louis, MO, USA)]. These cells were inoculated in T-75 cm^2^ flasks at a density of 2 × 10^5^ cells/cm^2^ in control DMEM and incubated at 37°C in a 5% CO_2_ saturated humid incubator. After 48 hours, the media was changed for the first time after washing twice with PBS to remove non-adherent cells, and then the media was changed every 3 days. When the cells approached confluence, the cell culture supernatant in the flask was discarded, the cells were washed two or three times with PBS, and 2.5 g/L trypsin/ethylene diaminetetraacetic acid (Gibco) was added for digestion. The cells were subcultured at a ratio of 1:3, and the third generation of cells was prepared for downstream-related experiments. The animal protocol used in the current study compliance with the relevant laws and institutional guidelines, and also was approved by the ethics review committee of the Third Xiangya Hospital, Central South University.

### Seeding of MSCs onto BCP and nHA-coated BCP scaffolds

Prior to cell culture, the BCP and nHA-coated BCP discs were sterilized in a low temperature plasma sterilizer (STERRAD 100S, Irvine, CA, USA). Immediately before cell seeding, the nHA-coated BCP and BCP scaffolds were soaked in osteogenic media [control media further supplemented with 10 nmol/L dexamethasone (Sigma), 200 μmol/L L-ascorbic acid-2-phosphate (Sigma), and 10 mmol/L β-glycerol sodium phosphate (Sigma)] [32] for 24 hours. MSCs were suspended in osteogenic media. Twenty thousand cells diluted into 2 ml osteogenic media were added to each well of a 24-well culture plate containing a BCP or nHA-coated BCP scaffold and maintained in a 5% CO_2_ humidified incubator at 37°C. The media was changed every 2 days.

### FESEM of MSCs attached to scaffolds

After culture of MSCs on BCP or nHA-coated BCP scaffolds for 1 day, samples were washed with PBS, fixed with 2.5% glutaraldehyde, dehydrated by an alcohol gradient, soaked in osmium tetroxide, dried, and coated with gold for observation by FESEM.

### Cell proliferation

After 1 day or 14 days, the media was removed and the cells cultured on BCP and nHA-coated BCP discs were washed with PBS. The samples were treated with the dye solutions of the LIVE/DEAD Cell Imaging kit (Molecular Probes, Eugene, OR, USA) for 15 minutes at room temperature for staining of live and dead cells. Green fluorescent live cells and red fluorescent dead cells were viewed by epifluorescence microscopy (IX71, Olympus).

Cell proliferation was calculated by measuring two parameters, the percentage of live cells (*P*_Live_) and cell attachment (*C*_Attach_), according to previously established methods [24,33]. Images were taken of three randomly selected fields of view for each sample (3 fields of view × 10 samples = 30 photographs per scaffold type), and live and dead cells were counted in each image. *P*_Live_ was calculated by dividing the number of live cells by the sum of the numbers of live and dead cells. *C*_Attach_ represents the number of live cells attached to the specimen and was calculated by dividing the number of live cells by the sample area. It is important to consider both *P*_Live_ and *C*_Attach_, because *P*_Live_ will be high if few dead cells are present even if only a small number of live cells are present. By contrast, *C*_Attach_ provides an absolute measure of cell survival on the scaffolds.

### MTT assay

Metabolic activity of the cells was analyzed using the MTT mitochondrial reaction, which is based on the ability of live cells to reduce a tetrazulium-based compound, MTT, to a purplish formazan product. Cell viability is proportional to the amount of dehydrogenase activity within the cells. After 14 days, BCP and nHA-coated BCP cell culture samples were transferred to new 24-well plates, washed twice with PBS, and incubated in 1 ml PBS and 100 μl MTT (5 mg/ml) solution at 37°C for 4 hours. The solution was then removed by aspiration, and 1 ml DMSO was added to completely dissolve the formazan. Two hundred-microliter samples of the final sample solutions were transferred to 98-well plates, and absorbance at 490 nm was measured with a microplate reader (Wallac Victor 31420 Multilabel Counter, Turku, Finland).

### Osteogenic differentiation of MSCs on BCP and nHA-coated BCP scaffolds

After 14 days of MCS culture on each scaffold type, the culture supernatant in each well was aspirated, and the samples were washed twice with PBS before transfer to a new 24-well plate. In the new plate, 0.2 ml de-ionized distilled water was added to each well before storage at –20°C overnight, followed by three freeze–thaw cycles (–80°C and room temperature for 30 minutes each) to disrupt the cell membrane. Samples were then transferred to 1.5-ml centrifuge tubes and subjected to 5000 *g* centrifugation at 4°C for 5 minutes before careful collection of the supernatant.

Three osteogenic markers were detected: alkaline phosphatase (ALP), collagen type I (Coll I), osteocalcin (OC). ALP activity, Coll I production, and OC production were quantified using the Rabbit Total Alkaline Phosphatase (TALP) enzyme-linked immunosorbent assay (ELISA) Kit, the Rabbit Collagen Type I, Col I ELISA Kit, and the Rabbit Osteocalcin/bone gla protein ELISA Kit (all from Sino-American Biotechnology, Wuhan, China), respectively, according to the manufacturer’s instructions. The absorbance of the solution in each well was measured using a microplate reader (Wallac Victor 31420 Multilabel Counter) at 450 nm, and the ALP, Coll I, and OC concentrations were calculated according to standard curves established based on the standard density as the abscissa and the optical density value as the ordinate, with MSCs cultured on tissue culture plates in a control media serving as a control.

### Statistical analysis

All data obtained are presented as mean ± SD values. One-way and two-way analyses of variance (ANOVAs) were carried out to detect significant effects of the experimental variables. Tukey’s multiple comparison tests were used with a *P* value of 0.05.

## Results

### Microstructure characterization of BCP and nHA-coated BCP scaffolds

**Figure 1:**
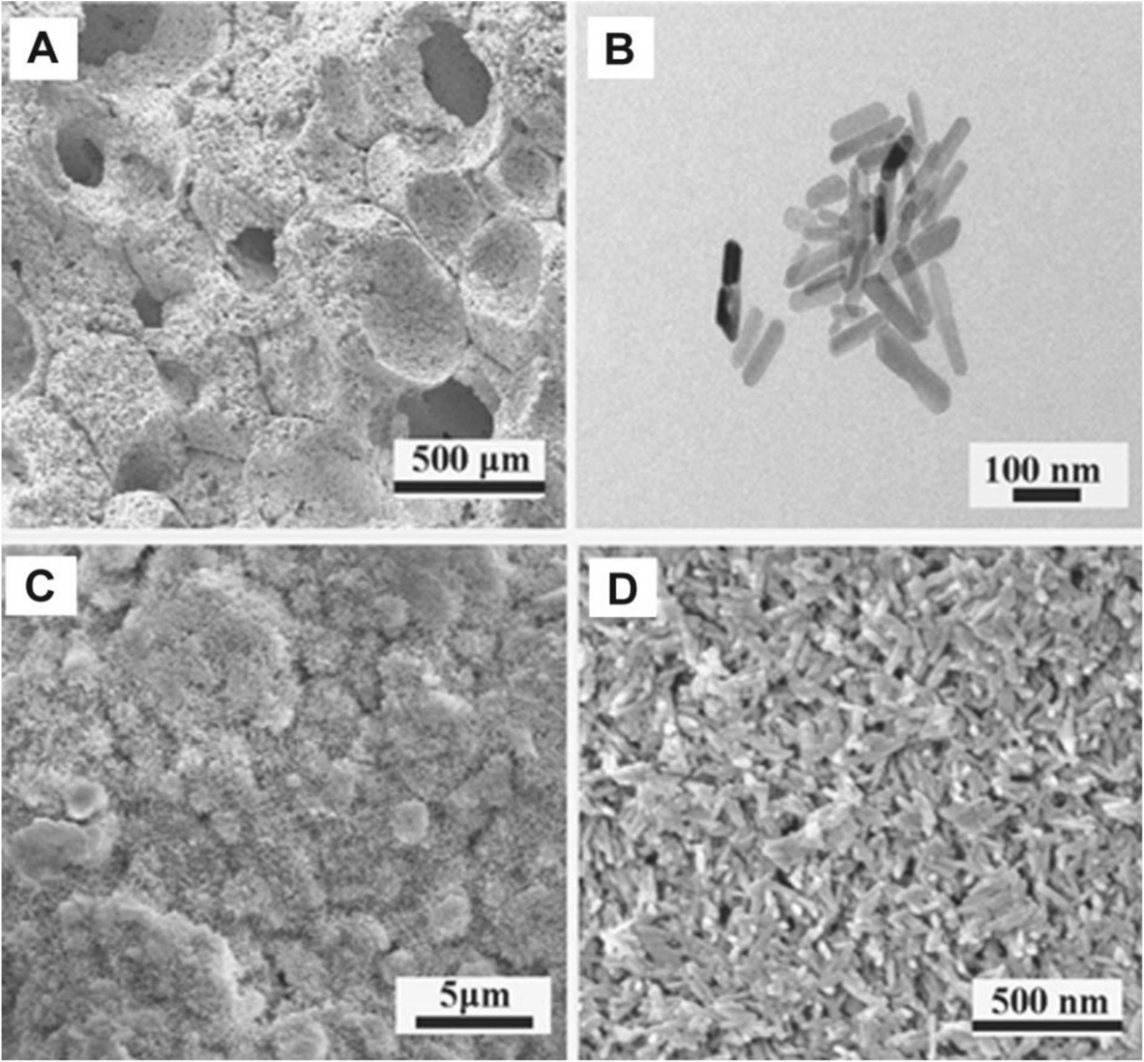
**Microstructure of the BCP scaffold, nHA powder, and the nHA-coated BCP scaffold. (A)** FESEM image of a polished surface of a BCP scaffold. **(B)** TEM micrograph of nHA powder. **(C)** Low magnification FESEM image displaying the formation of a nHA crystal deposition layer on the BCP scaffold surface. Similar nHA crystals were also observed on the walls of pores within the BCP scaffold. **(D)** High magnification image of the deposited nano-crystal layer.

Figure 1 shows the microstructures of a BCP scaffold, nHA powder, and a nHA-coated BCP scaffold. In images obtained by FESEM, macropores measuring from approximately 150 μm to almost 500 μm and containing many micropores (<10 μm) were observed in the BCP scaffolds (Figure 1A). The rod-like morphology observed in FETEM images of the nHA indicated that the material was effectively synthesized, with the length of individual crystals ranging from 50 to 200 nm and the width ranging from 15 to 30 mm (Figure 1B). The low magnification image in Figure 1C shows the nHA crystal layer deposited onto the BCP surface. Similar nHA crystals were also observed on the walls of pores within the BCP scaffold. The deposited layer consisted of nano-crystals, as shown in the higher magnification image in Figure 1D.

**Figure 2:**
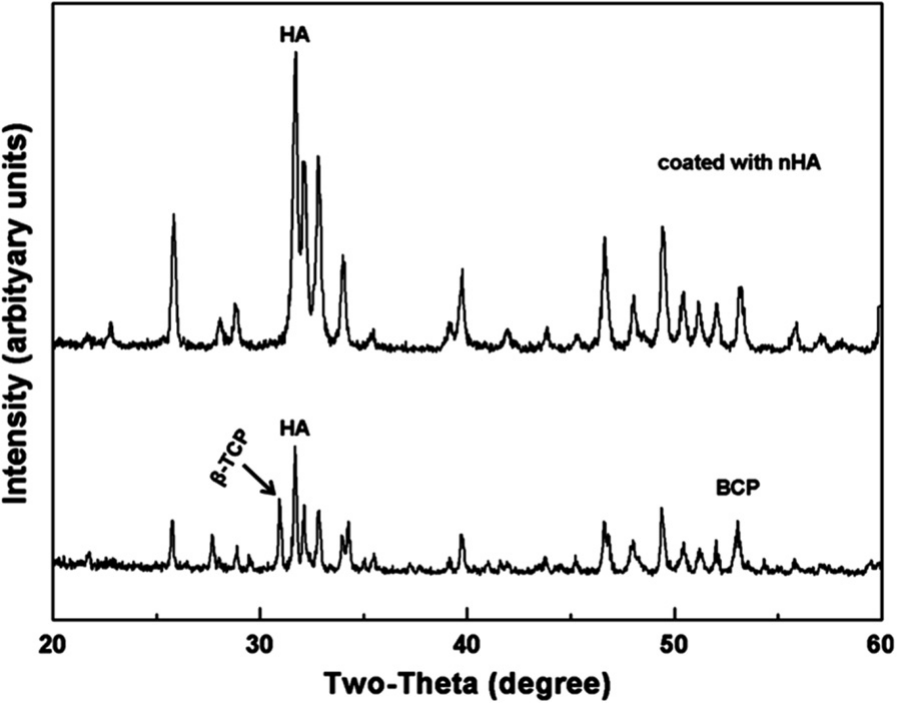
**XRD patterns for BCP and nHA-coated BCP ceramics.** The HA/TCP ratio was 65/35 without HA coating and 100/0 with HA coating.

XRD analysis of the BCP and nHA-coated BCP ceramic surfaces showed that the HA/TCP ratio was 65/35 on the BCP surface and 100/0 on the nHA-coated BCP surface (Figure 2), and the diffraction peak corresponding to the coating was consistent with the HA standard card (card no. 72-1243). These data indicate that the deposited HA formed a complete layer on the BCP surface.

The density of the BCP ceramic was 1.51 ± 0.03 g/cm^3^, and that of the nHA-coated BCP was 1.56 ± 0.04 g/cm^3^, with no significant difference (*P* > 0.1) between the densities of these two ceramics (n = 12). Similarly, the porosities of the two ceramics were not significantly different at 50.2% ± 1.1% and 48.7% ± 1.2% for BCP and nHA-coated BCP, respectively (*P* > 0.1; n = 12).

### Mechanical properties of BCP and nHA-coated BCP

The compressive strengths of BCP and nHA-coated BCP were 2.52 ± 0.22 MPa and 2.76 ± 0.18 MPa, respectively, and these measurements were not significantly different (*P* > 0.1) (n = 12). The flexural strengths of BCP and nHA-coated BCP were 1.85 ± 0.3 MPa and 1.95 ± 0.31 MPa, respectively, and these measurements also were not significantly different (*P* > 0.1; n = 12).

### MSC attachment to BCP and nHA-coated BCP scaffolds

**Figure 3:**
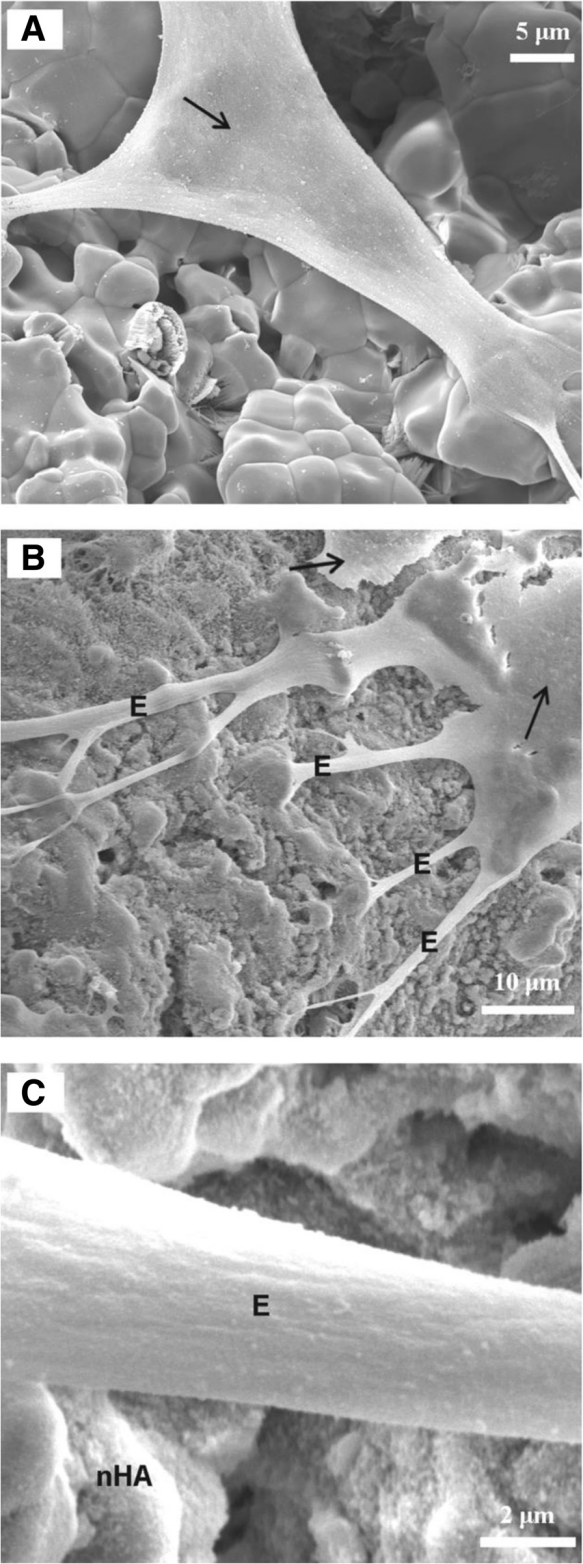
**FESEM images of MSCs adhered to BCP and nHA-coated BCP scaffolds.** Arrow in **(A)** indicates an MSC adhered to the BCP scaffold. **(B)** MSCs adhered to the nHA-coated BCP scaffold and developed long cytoplasmic extensions attached to the scaffold surface. **(C)** Cytoplasmic extensions (“E”) of an MSC adhered to the nHA crystals coating the BCP surface.

FESEM imaging showed an MSC (Figure 3A, MSC indicated by arrow) adhered to a BCP scaffold. On the nHA-coated scaffolds, MSCs also adhered and developed long cytoplasmic extensions (Figure 3B, extensions noted as “E”) attached to the scaffold surface. Figure 3C demonstrates how the cytoplasmic extensions (“E”) adhered to the nHA crystals coating the BCP surface.

### MSC proliferation and viability on BCP and nHA-coated BCP scaffolds

**Figure 4:**
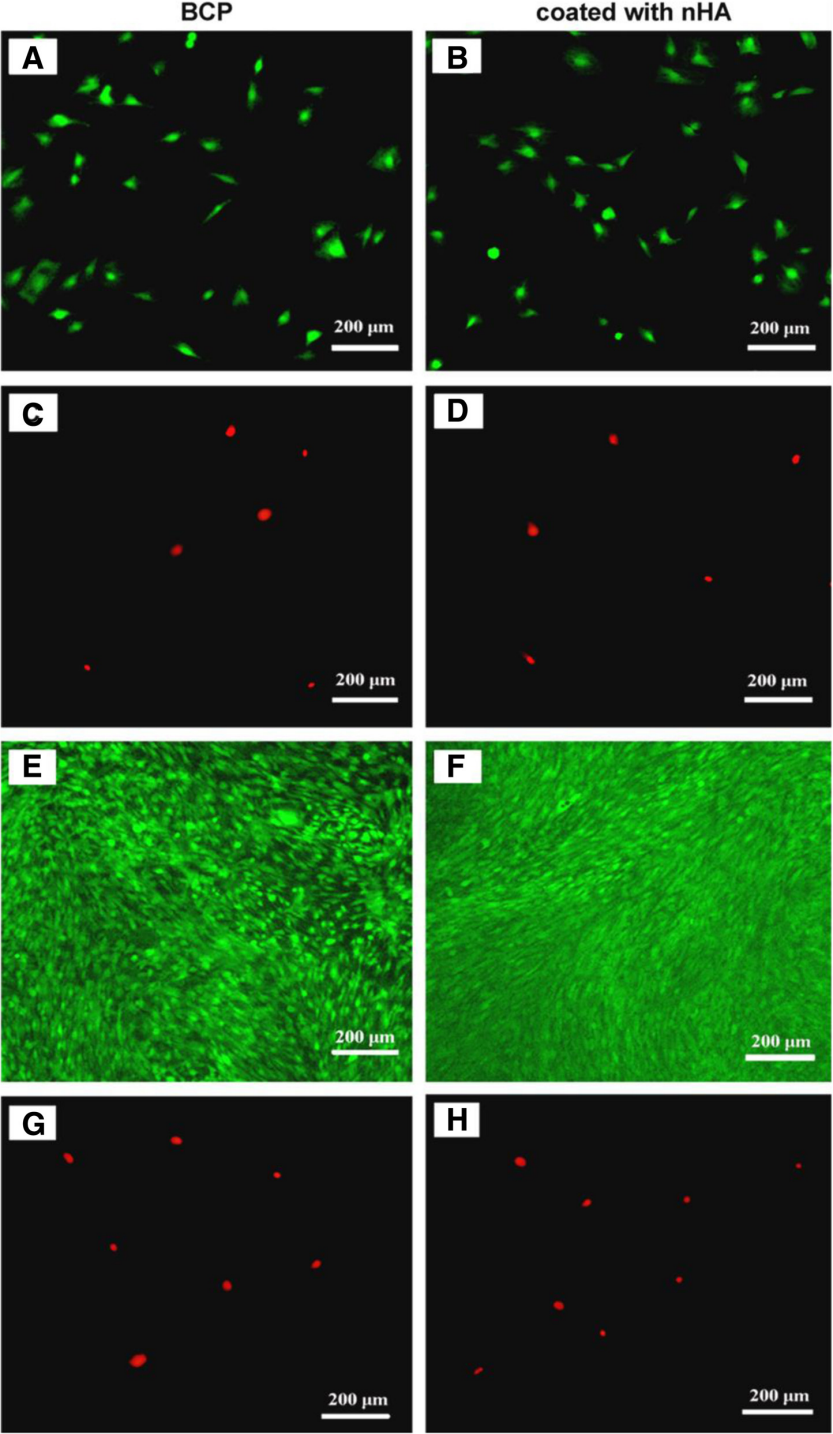
**Representative images of Live/Dead cell staining of MSCs cultured on BCP and nHA-coated BCP scaffolds.** After 1 day in co-culture, living cells (stained green) had adhered to both scaffold types, exhibiting normal polygonal morphology **(A, B)**, and few dead cells (stained red) were seen on either scaffold type **(C, D)**. After co-culture for 14 days, MSCs had proliferated on both scaffold types. MSCs have not yet reached full confluence on most regions of the BCP scaffolds **(E)**, but they had achieved a fully confluent monolayer on the nHA-coated BCP scaffolds **(F)**. After 14 days in culture, few dead cells were seen on either scaffold type **(G, H)**.

Representative images of fluorescently stained live and dead MSCs culture with BCP and nHA-coated BCP scaffolds after 1 and 14 days are shown in Figure 4. After the MSCs and scaffolds were co-cultured for 1 day, living cells (stained green) had adhered to both scaffold types and exhibited normal polygonal morphology (Figure 4A,B), and a few dead cells (stained red) were observed on the scaffolds (Figure 4C,D). After co-culture for 14 days, MSCs had proliferated on these scaffolds, reaching confluence on some areas, but not most, of the BCP scaffolds (Figure 4E) and achieving a fully confluent monolayer on the nHA-coated BCP scaffolds (Figure 4F). Although MSC proliferation was clearly observed on both scaffold types from days 1 to 14, after 14 days, the number of living cells on the nHA-coated BCP scaffolds was greater than that on the BCP scaffolds. After 14 days, the number of dead cells on either scaffold type remained low (Figure 4G,H).

**Figure 5:**
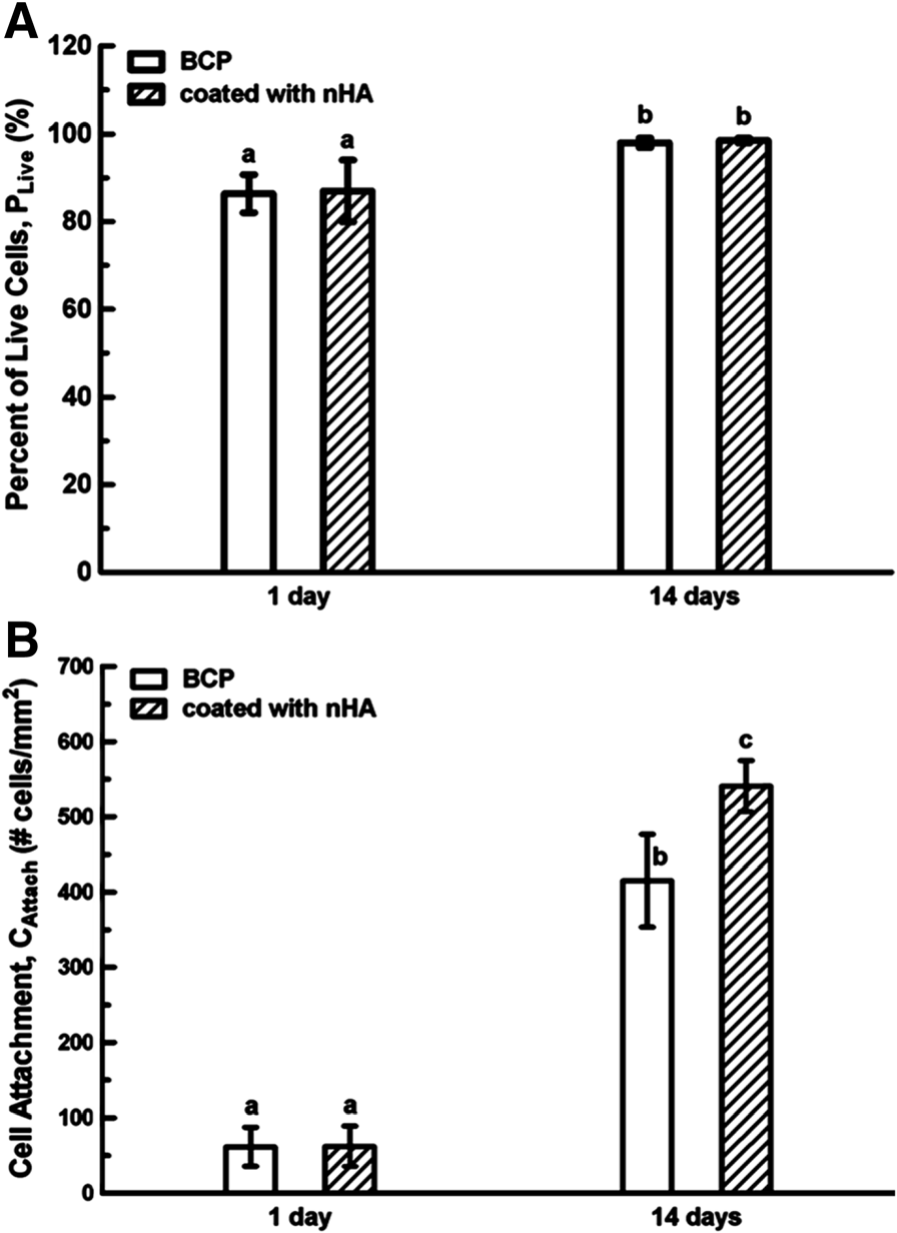
**MSC proliferation on the BCP and nHA-coated BCP scaffolds. (A)** Percentage of living cells, *P*_Live_. **(B)** Attachment density, *C*_Attach_. *P*_Live_ did not differ significantly between the BCP and nHA-coated BCP groups at either day 1 or day 14, but did increase significantly from day 1 to day 14 on both scaffold types. From day 1 to day 14, *C*_Attach_ of MSCs on BCP and nHA-coated BCP scaffolds increased 5.7- and 7.6-fold, respectively. Data are given as mean ± SD, and n = 12 with dissimilar letters indicating a significant difference (*P* < 0.05).

The percentages of live MSCs, *P*_Live_, cultured with BCP and nHA-coated BCP scaffolds for 1 day and 14 days were plotted (Figure 5A). After 1 day, the *P*_Live_ of the BCP group was 86.4 ± 4.4%, and that of the nHA-coated BCP group was 87 ± 7.1%, with no significant difference between the two materials (*P* > 0.05). After 14 days, the *P*_Live_ of the BCP group was 98 ± 1.1%, and that of the nHA-coated BCP group was 98.5 ± 0.5%, also with no significant difference between the two materials (*P* > 0.05). However, compared to the values on day 1, the *P*_Live_ values for both groups significantly increased by day 14 (*P* < 0.05). Figure 5B shows the attachment results for MSCs cultured with BCP and nHA-coated BCP scaffolds after 1 and 14 days. *C*_Attach_ for BCP increased from 62 ± 26 cells/mm^2^ after 1 day to 415 ± 62 cells/mm^2^ after 14 days (*P* < 0.05). For nHA-coated BCP, *C*_Attach_ increased from 63 ± 27 cells/mm^2^ after 1 day to 541 ± 35 cells/mm^2^ after 14 days (*P* < 0.05). After 14 days, *C*_Attach_ on nHA-coated BCP (541 ± 35 cells/mm^2^) was higher than that on BCP (415 ± 62 cells/mm^2^) (P < 0.05). The results of the MTT assay of MSC viability did show a significant difference between the two scaffold types, with absorbance values of 1.00 ± 0.13 for the BCP group and 1.13 ± 0.09 for the nHA-coated BCP group (*P* < 0.05) (n = 12).

Together, these data indicate that the nHA coating promoted MSC proliferation and increased MSC viability.

### ALP activity, Coll I production, and OC production of MSCs on BCP and nHA-coated BCP scaffolds

**Figure 6:**
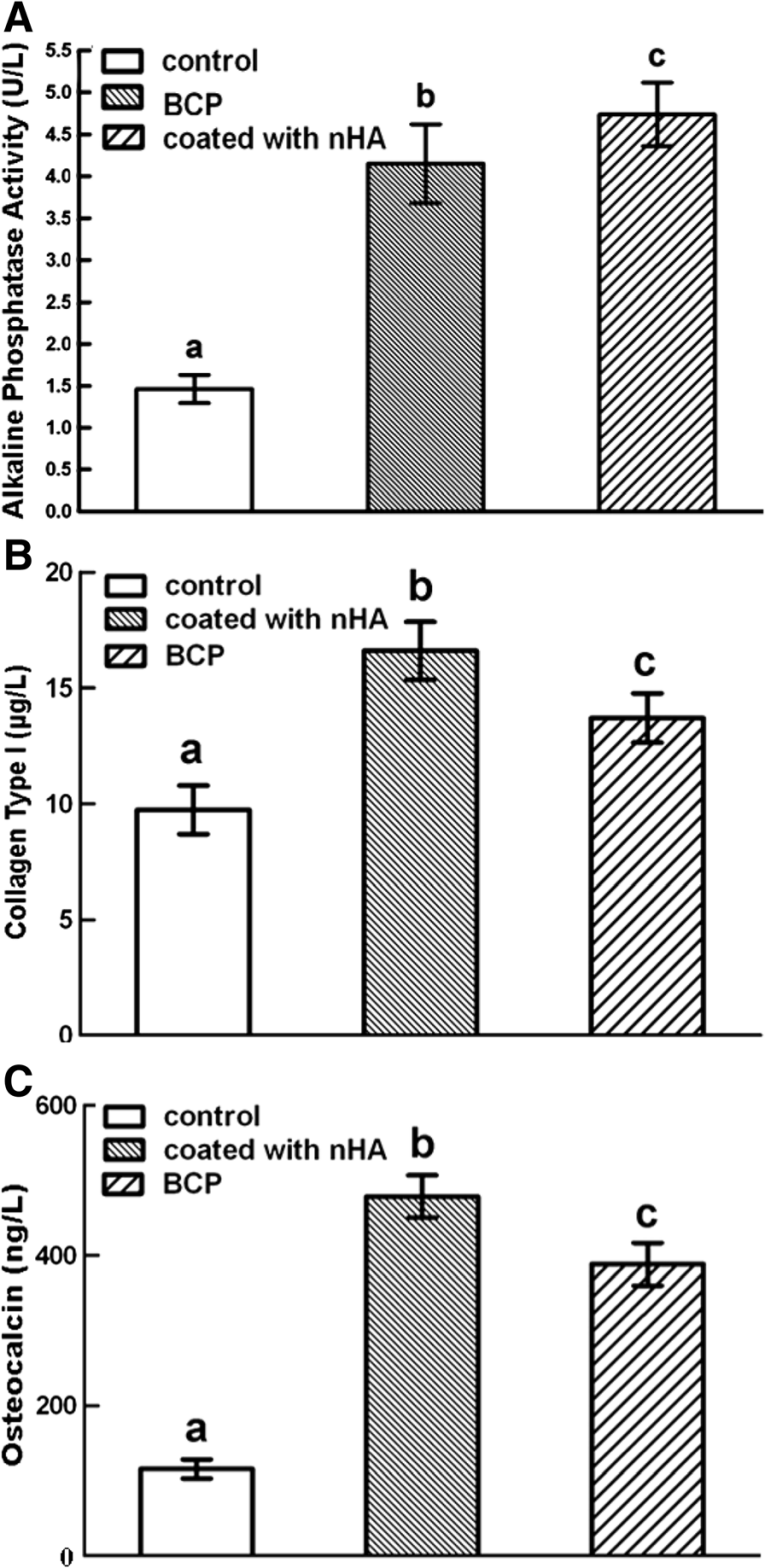
**Osteogenic differentiation of MSCs cultured on BCP and nHA-coated BCP scaffolds. (A)** ALP activity, **(B)** Coll I production, and **(C)** OC production. MSCs on nHA-coated BCP scaffolds produced higher levels of these osteogenic markers than MSCs on BCP scaffolds (*P* < 0.05), and the levels of ALP, Coll I, and OC production on both scaffold types were significantly greater than those of the control group. Data are given as mean ± SD, n =12 with dissimilar letters indicating a significant difference (*P* < 0.05).

As important markers of osteogenic differentiation [34], ALP activity, Coll I production, and OC production were measured for MSCs on BCP and nHA-coated BCP scaffolds (Figure 6). The ALP concentration (Figure 6A) measured for MSCs on nHA-coated BCP scaffold samples was 4.74 ± 0.38 U/L, which was significantly greater (*P* < 0.05) than that for MSCs on BCP scaffold samples (4.15 ± 0.42 U/L). The ALP concentrations measured for both the BCP and nHA-coated BCP groups were significantly greater than that of the control group (1.46 ± 0.17 U/L; *P* < 0.05) (n = 12). The Coll I concentration (Figure 6B) measured for MSCs on nHA-coated BCP scaffold samples was 16.60 ± 1.25 μg/L, which was significantly greater (*P* < 0.05) than that for MSCs on BCP scaffold samples (13.71 ± 1.06 μg/L). The Coll I concentrations measured for both the BCP and nHA-coated BCP groups were significantly greater than that of the control group (9.74 ± 1.05 μg/L; *P* < 0.05) (n = 12). The OC concentration (Figure 6C) measured for MSCs on nHA-coated BCP scaffold samples was 478.04 ± 28.27 ng/L, which was significantly greater (*P* < 0.05) than that for MSCs on BCP scaffold samples (388.02 ± 30.49 ng/L). The OC concentrations measured for both the BCP and nHA-coated BCP groups were significantly greater than that of the control group (115.53 ± 12.49 ng/L; *P* < 0.05) (n = 12).

## Discussion

Scaffold selection is a key point in bone tissue engineering, and porous BCP ceramics offer many of the properties of an ideal scaffold for bone tissue regeneration as summarized by Porter et al. [2]. Specifically, porous BCP ceramics are able to: “1) provide temporary mechanical support to the affected area; 2) act as a substrate for osteoid deposition; 3) contain a porous architecture to allow for vascularization and bone in-growth; 4) encourage bone cell migration into the scaffold; 5) degrade in a controlled manner to facilitate load transfer to developing bone; 6) produce non-toxic degradation products; 7) not incite an active chronic inflammatory response; and 8) be sterilized without loss of bioactivity [2,10-12]”. However, porous BCP ceramics lack the ability to support and promote osteogenic differentiation of bone precursor cells within an implanted scaffold [11,13], and therefore, this material is not able to induce satisfactory bone formation and its clinical use has been limited [9-11,14].

In this study, we coated conventional porous BCP ceramics with nHA to improve the material’s osteoinductive potential and create a new bioresorbable ceramic scaffold for application in bone tissue engineering. We found that nHA coating of BCP scaffolds had little impact on the porosity, density, and flexural and compressive strength of BCP. A deposited layer of rod-like nHA was formed on the BCP surface and pore walls, completely covering the conventional porous BCP ceramic surface. Thus, the hydrothermal deposition method used in this study was able to form a coating layer of nHA on the BCP surface that did not significantly change the physical and mechanical properties of BCP. In addition, the hydrothermal deposition method used in this study is simple, quick, and does not require synthesis of a large amount of nHA to coat the conventional porous BCP ceramics, which reduces material preparation costs. Even more importantly, the biological characteristics of nHA-coated BCP are similar to those reported for nanostructured BCP [9,23].

To further investigate the osteoinductive potential of nHA-coated BCP ceramics, we seeded MSCs on BCP and nHA-coated BCP scaffolds and compared cell adhesion, proliferation, and ALP activity on these two scaffold types. After 1 day in culture, MSCs had attached and stretched, exhibiting normal cell morphology on both scaffold types. However, on the nHA-coated BCP scaffolds, MSCs stretched more obviously than on the BCP scaffolds. MSC spreading and development of cytoplasmic extensions indicated good attachment and viability of MSCs on both materials. MSCs greatly proliferated from day 1 to 14 in culture, increasing in number by 5.7-fold on the BCP scaffolds and 7.6-fold on the nHA-coated BCP scaffolds, and the number of living cells on the nHA-coated BCP scaffolds was greater than that on the BCP scaffolds. Furthermore, MTT assay results showed that cell viability on the nHA-coated BCP scaffolds was higher than on the BCP scaffolds. Therefore, although both scaffolds can support the adhesion and proliferation of MSCs over time in culture, the nHA-coated BCP scaffolds supported better MSC proliferation, indicating that the nHA coating on the BCP scaffold surface can promote MSC adhesion, proliferation, and viability, consistent with previous studies that examined bone-related cell behavior on nHA [17-21,35]. For example, Zandi et al. [20] reported that coating of HA/gelatin scaffolds with nHA enabled better MSC attachment and proliferation in comparison with noncoated scaffolds. Chen et al. [21] reported that after a nHA layer was coated onto a roughened titanium surface, the proliferation of MSCs was higher than on the corresponding noncoated titanium surfaces. A novel composite coupling nHA and poly [4-methacryloyloxyethyl trimellitate anhydride (4-META)]-grafted silk fibroin (SF) described by Korematsu [35] could improve L-929 cell adhesion and bioactivity compared to the original SF due to a coating of sintered nHA on 4-META -grafted SF.

In addition, the osteogenic markers ALP, Coll I, and OC were expressed by MSCs cultured on the BCP and nHA-coated BCP scaffolds, but MSCs on the nHA-coated BCP scaffolds produced more ALP, Coll I, and OC than MSCs on the BCP scaffolds. Based on these data, it seems that the high numbers of attached MSCs, as well as their osteogenic differentiation on nHA-coated BCP scaffolds, were the result of the surface properties introduced by nHA to the scaffold material’s surface. A possible mechanism responsible for these effects on MSC proliferation and osteogenic differentiation may be the small scale of the scaffold surface nanostructure, which enhanced interfacial adhesion of cells to nHA. The nanoscale surface structure gives the BCP much greater surface area and reactivity, which in turn enhances the scaffold’s ability to guide cell mitogenic behavior [19,20,36,37].

In addition to the scale of the nHA surface structure, the crystallinity of nHA particles also affects cell behavior, with the surface chemistry and topography of lower crystallinity being favorable for cell attachment and differentiation [19,38]. The results of our study further support this notion. Another possible mechanism responsible for the increased MSC proliferation and ALP activity on nHA-coated BCP scaffolds is that the nHA coating was partially dissolved to release Ca^2+^ ions. Appropriate Ca^2+^ concentrations have been shown to favor cell proliferation and differentiation [19,39]. Therefore, the nHA powder synthesized by a wet chemical method is suitable for use as a coating material on porous BCP, and nHA shows more similarity in terms of morphology and crystal structure to natural apatite [Figure 1(B), and Figure 2 (coated with nHA)]. Further studies should investigate the mineralization of MSCs on nHA-coated BCP scaffolds and the osteoinductivity of nHA-coated BCP *in vivo.*

## Conclusions

In this study, for the first time, we coated porous BCP ceramic scaffolds with nHA and then induced the osteogenic differentiation of rabbit bone marrow-derived MSCs seeded on these scaffolds. The hydrothermal deposition method used to apply nHA to the BCP scaffolds formed a dense layer of rod-like nHA crystals on the BCP surface and on the pore walls. The nHA coating did not significantly affect the density, porosity, flexural strength, or compressive strength of the underlying BCP. Our results demonstrate that BCP scaffolds coated with nHA were more conducive for MSC adhesion, proliferation, and osteogenic differentiation than conventional, uncoated BCP scaffolds, indicating that nHA coating can enhance the osteoinduction potential of BCP ceramics, making this material more suitable for applications in bone tissue engineering.

## Competing interests

The authors have no competing interests to declare.

## Authors’ contributions

YZ conceived the project and revised the manuscript. JH carried out the microstructure characterization and the Live/Dead cell staining and drafted the manuscript. LH performed the mechanical testing of the specimens, ALP measurement, and revised the manuscript. JL carried out the FESEM and MTT assay. HL carried out the isolation and culture of bone marrow MSCs and helped to revise the manuscript. All authors read and approved the final paper.

## Pre-publication history

The pre-publication history for this paper can be accessed here:

http://www.biomedcentral.com/1471-2474/15/114/prepub
